# Advancing Malaria Vector Control: Insights Into Mosquito Immunity and Genetic Strategies

**DOI:** 10.1155/tswj/7634044

**Published:** 2026-04-17

**Authors:** Wisdom D. Cleanclay, Eniola D. Adedoyin, Suleiman Zakari, Olubanke O. Ogunlana, Emeka E. J. Iweala, Shalom N. Chinedu

**Affiliations:** ^1^ Department of Biochemistry, Covenant University, Ota, Ogun, Nigeria, covenantuniversity.edu.ng; ^2^ Covenant Applied Informatics and Communication Africa Centre of Excellence, Covenant University, Ota, Ogun, Nigeria, covenantuniversity.edu.ng; ^3^ Department of Biochemistry, College of Medicine, Federal University of Health Sciences Otukpo, Otukpo, Benue, Nigeria, medcol.mw

**Keywords:** genetic approaches, malaria, mosquito immunity, *Plasmodium* parasites, vector control

## Abstract

Malaria remains a major global health challenge, particularly in sub‐Saharan Africa where *Anopheles* mosquitoes transmit the *Plasmodium* parasites. Resistance to insecticides remains an obstacle in spite of the efforts to control malaria vector. The interaction between *Plasmodium* parasites and mosquito vectors, with a focus on the immunity of mosquitoes and approaches to combat malaria, is examined in this review. This review explores the potential of genetic approaches including CRISPR‐Cas9, *Wolbachia*, RNA interference (RNAi), and symbiont‐based strategies for the control of malaria vector. The innate immune system of *Anopheles* mosquitoes that identify, recognize, and limit *Plasmodium* infection through pathogen recognition receptors, signaling pathways, and effector mechanisms like antimicrobial peptides and melanization is well developed. However, *Plasmodium* has developed several evasion mechanisms to establish infection. This led to various genetic modification techniques being designed to reduce vector population and transmission. Gene drive such as CRISPR‐Cas9 can introduce genetic alterations to interfere with the transmission of malaria; *Wolbachia* interferes with vector competence, RNAi‐mediated gene to target relevant genes involved in reproduction and survival. Self‐limiting strategies such as RIDL and pgSIT genetically modified insect releasement to the environment. mosGILT is an emerging immune regulator which has shown relevance in blocking transmission. This review explores the potential of these genetic approaches in malaria vector control efforts, highlighting their advantages and imitations. Further research should explore mosquito immune genes and pathways in developing innovative and acceptable genetic vector control approaches.

## 1. Introduction

Malaria, a life‐threatening disease caused by *Plasmodium* parasites, remains a major global health challenge, with an estimated 282 million cases and 610,000 deaths in 2024 [1]. The burden of malaria immensely affects vulnerable populations in sub‐Saharan Africa, where *Anopheles* mosquitoes serve as the vectors responsible for transmitting the parasite to humans [2]. In spite of remarkable progress in malaria control efforts, which include the global deployment of insecticide‐treated bed nets and artemisinin‐based combination treatment, the emergence and spread of insecticide resistance among mosquito populations present a significant challenges to malaria elimination efforts [3]. The parasites’ ability to evade antimalarial drugs’ effect has been associated with the drug resistance [4]. Controlling the vector that acts as the main host of the *Plasmodium* parasite has been the goal of several control initiatives in the sub‐Saharan African area [5]. Alternative strategies such as genetic techniques use mosquitoes’ genetic composition for vector control, thereby making mosquitoes less vulnerable to *Plasmodium* infection [6].

The vitality of malaria transmission is relative to the interaction between mosquito vector and the *Plasmodium* parasite [7]. The innate immune system of *Anopheles* mosquitoes allows them to recognize, neutralize, and eliminate *Plasmodium* parasites [8]. At the molecular level, mosquito immunity has an effect on vector competence [9], the possibility of mosquitoes to be infected and transmit malaria parasites which present new approaches to disrupt the cycles of malaria transmission.

Transgenesis, which is genetic alteration approach, and CRISPR/Cas9, a gene editing mechanism, are promising approaches for designing improved mosquitoes that are resistant to *Plasmodium* parasites [10]. Sterile insect technique (SIT), which is a genetic approach [11], and gene drive systems give novel opportunities to control mosquito populations and put a stop to the transmission of malaria from the source. There is the emergence of functional genomics, a high‐powered tool for dissecting mosquito immune response to malaria at the molecular level [12]. RNA‐seq and genome editing techniques like CRISPR‐Cas9, which are advanced sequencing methodologies, have been useful in comprehensive profiling and modification of the pathways and immune genes of mosquitoes [13, 14]. Combined methods of proteome and metabolomics have improved the understanding of the interaction between parasite growth, parasite–vector relationship, and mosquito immune responses [15, 16].

This review examines the genetic approaches for malaria vector control and immunity of mosquito to plasmodium. It also recognizes the opportunities and limitations of malaria vector control strategies by examining previous research and highlighting the area of improvement for efficient malaria control strategies in relations to the goal of global health.

### 1.1. Mosquito Immunity as a Target for Genetic Malaria Control

The innate immune system in *Anopheles* mosquitoes is well established and is crucial to the determination of vector competence and the efficiency if malaria transmission [17, 18]. Cuticle and midgut epithelium, cellular responses, and humoral effector mechanisms are collective physical barriers that the immune system uses to recognize and resist plasmodium infection [19]. Pattern recognition receptors (PRRs) initiate the mosquito immunity at the molecular level, recognize parasites‐derived molecules, and trigger subsequent signaling pathways [20]. Thioester‐containing proteins, fibrinogen‐related proteins, peptidoglycan recognition proteins, and Gram‐negative bacteria–binding proteins are important PPRs that work together to interfere in parasite recognition and activation of the immune system [21].

**FIGURE 1:**
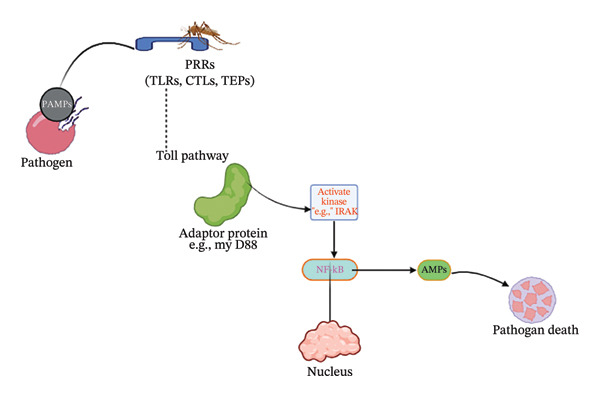
The signaling pathway of mosquito’s innate immunity: The innate immune response in the mosquito is triggered by PRRs that detect the presence of a pathogen, and this event leads to a series of signaling cascades downstream. The Toll pathway triggers transcription factors such as NF‐κB and the expression of various immune effectors like antimicrobial peptides (AMPs).

The involvement of these receptors, especially the Toll and immune deficiency (IMD) pathways, stimulates the sequence of chemical reactions that results into transcriptional stimulation of immune effector genes [22]. The production of antimicrobial peptides, such as defensins and cecropins, as well as complement‐like factors that specifically target invading parasites is stimulated by these pathways. Likewise, cellular immune responses including phagocytosis, melanization, and nitration further restrict parasite development within the mosquito midgut and hemocoel (Figure 1) [23–25].

During malaria infection, *Anopheles* mosquitoes mount stage‐specific immune responses that significantly limit *Plasmodium* survival [26–28]. Parasite killing occurs primarily during the ookinete stage, where immune effectors and midgut‐associated responses act as a major transmission bottleneck [29–32]. Thioester‐containing protein 1 (TEP1), fibrinogen‐related proteins, and clip‐domain serine proteases are complementary proteins that are important in the recognition and clearance of parasites [24, 33]. However, *Plasmodium falciparum* still possesses immune evasion mechanisms to develop in the mosquito vector in spite of these defenses. Mosquito immune activation is suppressed by the parasite surface protein *Pf*s47 through the inhibition of JNK‐mediated apoptosis and obstructing midgut nitration responses, which enables parasite survival [34–38]. The interdependent interaction between mosquito immune defenses and the evasion mechanisms of parasite is as a result of the robust host–parasite interaction.

The identification of immune genes and pathways that restrict parasite development has direct implications for genetic malaria control. Immune effectors such as TEP1, fibrinogen‐related proteins, antimicrobial peptides, and regulators of myelinization have emerged as promising targets for genetic intervention aimed at reducing vector competence [39–41]. CRISPR/Cas9‐mediated manipulation of immune genes has been used to alter mosquito susceptibility to *Plasmodium* infection [41]. For instance, knock out of fibrinogen‐related proteins 1 (FREP1) in *Anopheles gambiae* significantly suppresses parasite development without imposing severe fitness costs on the mosquito. Similarly, the modulation of complement‐like pathways involving TEP1 and associated immune regulators has been shown to substantially reduce parasite survival in the midgut. These shows that immune gene manipulation can generate stable parasite‐refractory phenotypes [42, 43].

Beyond gene disruption, immune gene manipulation has been translated into vector control applications through the engineering of transgenic mosquitoes with enhanced resistance to malaria parasites [44]. Early transmission‐blocking strategies focused on constitutive expression of antiparasitic effector molecules, including antimicrobial peptides and complement‐like factors [44, 45], including oocyst maturation and sporozoite invasion of the salivary gland [46] to disrupt parasite development at critical stages. More recent approaches integrate immune‐based transgenes with gene drive systems, enabling the efficient spread of parasite‐refractory traits through mosquito populations, as demonstrated in gene‐drive strains carrying anti‐*Plasmodium* effector molecules in *Anopheles* mosquitoes [47]. Such population‐replacement strategies aim to suppress malaria transmission without necessarily reducing mosquito population size, offering a potentially sustainable and species‐specific intervention [42, 48, 49].

In addition to canonical immune effectors, emerging regulators such as mosquito gamma‐interferon‐inducible lysosomal thiol reductase (mosGILT) have been identified as important modulators of mosquito immunity and reproductive fitness. Gene‐edited mosquitoes lacking a gamma‐interferon‐inducible lysosomal thiol reductase‐like protein (*mosGILT*) have lower *Plasmodium* infection, which is linked to impaired ovarian development and immune activation. The loss of mosGILT has been shown to reduce *Plasmodium* vulnerability in experimental studies while compromising germ cell development at the same time, especially in female mosquitoes [50]. The alterations in immune and metabolic‐related genes in various tissues such as fat bodies and ovaries are revealed by transcriptome profiling. This has shown the potential of mosGILT as a transmission blocking target approach that merges enhanced immune refractoriness with reduced vector fitness [50].

The combination of mosquito immune biology with genome engineering technologies provides a coherent approach for malaria vector control.

### 1.2. Innovative Strategies for Malaria Vector Control

The strategies of gene drive used to modify the genetic composition of mosquito are a great avenue to reduce the potential of malaria parasite transmission within the mosquito population [51]. Vector‐borne disease can be controlled by rightly suppressing the vector population or expanding the genetic payloads developed to reduce the transmission of pathogens using gene drive [52]. Biasing the inheritance of the gene drive construct is the means in which gene drive technology operates, which allows it to be transmitted to a more proportion of offspring than it would have been under the Mendelian inheritance. As a result of this bias inheritance, the interrelated genetic trait linked to the drive spread [53] and through the target population, genetic traits linked to the drive can spread swiftly.

There are two broad classifications of gene drives used for vector control. They include the population modification and the population suppression. The population modification drives are developed to spread the nucleic acid cargo that hinders the development of the parasite or elevate the immune responses of the mosquito to reduce vector competence [54, 55]. However, the population suppression gene drives bias inheritance in such a way that the mosquito population size is mitigated majorly by interrupting the genes that are responsible for female fertility, determination of sex, or viability. There can be great achievement and sustained decrease in *Anopheles* populations using suppression drive where there can be propagation of the drive element in spite of imposing a fitness cost. Both classifications of drives are important for genetic strategies that are currently examined for the control of malaria [56].

A powerful CRISPR‐Cas9‐based gene drive technology for malaria vector control has emerged, as this approach uses the genetic modification that can effectively spread via the population [54]. CRISPR‐Cas9 system triggers the cleavage of targeted DNA at a specific locus, and the gene drive cassette is duplicated into the homologous chromosome during DNA repair in the homing‐based gene drives. There are, therefore, disruption and modification of target genes as well as the construction of the gene drive itself to propagate through populations due to the bias inheritance, resulting in population suppression or replacement approaches.

The induction of lethality or sterility in offsprings for the suppression of mosquito population is an application of gene drive technology in malaria vector control [57]. The introduction of construct that encodes lethal or sterile genes in the mosquito populations allows for the reduction and limitation of the numbers of mosquitoes to transmit malaria parasites [56]. Genetic alterations that confer resistance to malaria parasite in a mosquito population can be achieved using gene drive technology [58, 59]. Depending on various purposes, modification drives can be used for the inactivation of transgenic host factor genes, the manipulation of miRNAs and lncRNAs, and the expression of antiparasitic effector genes in a population. Any of these strategies can yield into effective suppression or replacement of existing population, thereby hindering the transmission of malaria parasites. The population suppression approach employs techniques that lead the targeted population to elimination, and this approach focuses on targeting genes or chromosomes and interrupting their functionality [54, 57]. Malaria transmission cycle can be hindered by using a gene drive system that target genes responsible for parasite–mosquito interactions such as immune‐related pathways or receptors by engineering mosquitoes with high immunity to the parasites [59]. Gene drives that can also disrupt the parasites’ life cycle or that increase the mosquito resistance to infection can be engineered with mosquitoes to reduce the transmission of malaria [60].

Gene drive technology, despite its potential for malaria control, faces several challenges, including the emergence of resistance alleles in mosquito populations that can compromise the effectiveness of suppression strategies [57, 58]. Resistance can arise from error‐prone DNA repair mechanisms that generate resistant alleles and reduce the effectiveness of gene drive suppression strategies. This challenge can be addressed by developing gene drives that contain multiple gRNA target sites to reduce the emergence of resistance alleles. Recently, the optimization of gene drive architecture has been demonstrated for *Anopheles stephensi* suppression systems [52].

### 1.3. Symbiont‐Based Approaches

**FIGURE 2:**
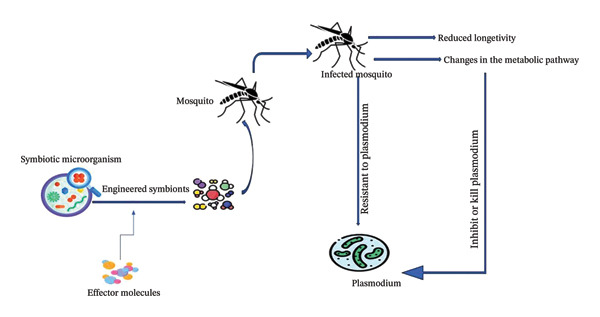
Symbiotic organisms genetically modified for the management of mosquito populations.

Malaria vector can be controlled by targeting symbiotic microorganisms in the mosquito hosts, which is a promising target by altering the physiology, immunity, and vector competence of the mosquito [61]. Symbiont‐based approaches involve leveraging the interactions between mosquitoes and their endosymbiotic microbes to manipulate vector traits relevant to malaria transmission (Figure 2) [62, 63].

Symbiont‐based mosquito control has the ability to reduce resistance while minimizing environmental impact. Symbionts can be used for vector control in two ways: (i) directly delivering natural symbionts into mosquitos to disrupt their physiology and reduce vector competence or (ii) genetically modifying symbionts to express antipathogen effector molecules and then delivering the engineered symbiont into mosquitos to reduce vector competence or vectorial capacity [55]. It is the use of engineered symbiotic bacteria to deliver antiparasitic molecules or immune‐stimulatory factors to mosquitoes [21]. Molecules that disrupt parasite development or enhance the immune response to parasites and vector competence can be produced by engineering symbiotic bacteria to reduce or limit the transmission of malaria [55]. This approach needs a proper study of the mosquito microbiome, the interaction with the host, and the implications of manipulating the symbiotic communities [64]. Also, the consistency and persistence of engineered symbiont strains within mosquito populations need to be carefully evaluated to ensure their long‐term effectiveness in malaria control efforts [55].

### 1.4. *Wolbachia*‐Based Strategies

The potential to manipulate mosquito reproduction using Wolbachia, a bacterial symbiont within the cell that induces cytoplasmic incompatibility, has been studied as a strategy to reduce the transmission of pathogens [65]. In a mosquito population, *Wolbachia* can spread via maternal inheritance, thereby resulting in the replacement of the population. The ability of mosquito to spread malaria parasite has been reduced through *Wolbachia* infection, which is promising for malaria control [66]. Due to its rigorous experimental characterization and ongoing field evaluation, this approach is considered distinct from other strategies and represents a less‐studied symbiont‐based approach.

**FIGURE 3:**
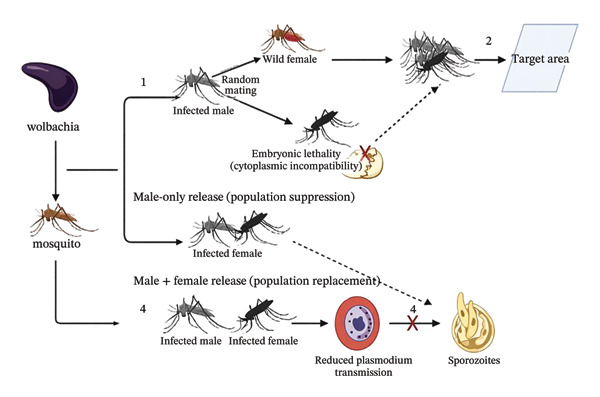
*Wolbachia*‐based strategies for malaria transmission blockade.

*Wolbachia*, a genus of bacteria naturally found in mosquitoes, has shown promise as a tool for controlling malaria vectors [67]. Different phylogenetic strains of *Wolbachia* induce distinct extended phenotypes in the mosquito they infect; the effect induced by this bacterium in their host can be cytoplasmic compatibility, incompatibility, or compatibility in only one direction. *Wolbachia*‐based strategies involve introducing *Wolbachia*‐infected mosquitoes into target populations to disrupt their ability to transmit malaria parasites [68]. This approach has the potential to reduce vector competence and interrupt malaria transmission cycles (see Figure 3).

*Wolbachia-*based interventions can be implemented using two distinct approaches: population suppression through the release of infected males only, which is self‐limiting and analogous to the SIT, or population replacement through the release of both infected males and females, allowing *Wolbachia* to spread and reduce mosquito vector competence [69].

*Wolbachia-*infected male mosquitoes mate randomly with wild‐type females. When only infected males are released, cytoplasmic incompatibility results in embryonic lethality, leading to self‐limiting population suppression analogous to the SIT. However, when both *Wolbachia*‐infected males and females are released, this results into consistent maternal transmission of *Wolbachia*, which allows the spread of the infection in the population. With this replacement approach, there is reduction in mosquito vector competence and limitation of parasite development resulting in the decrease of malaria transmission.

When mosquitoes that are infected with *Wolbachia* are released, they, therefore, disrupt or hinder the spread of the disease either by reducing the population density of the mosquito through the cytoplasmic incompatibility which is induced by embryonic lethality or the impairment of the development of the pathogen [65]. Studies have shown that *Anopheles* are really infected by *Wolbachia* and in African populations, several strains have been identified to support these findings transmission [42, 70].

The efficiency of *Wolbachia* in reducing malaria transmission has been seen in several studies. There is a reduction in the susceptibility of *Plasmodium falciparum* infection when *Anopheles stephensi* is infected with *Wolbachia*, thereby leading to a decrease in the parasite transmission potential [71]. There is also a reduction in malaria transmission in *Anopheles gambiae* mosquitoes infected with *Wolbachia* in field settings [72]. This approach comes with its own challenges, which includes the optimization of *Wolbachia* strain selection and the establishment of stable infections in mosquito populations [73]. If properly addressed, this approach can be considered to maximize vector management as integrated strategies with other control measures [65].

### 1.5. RNA Interference (RNAi)

**FIGURE 4:**
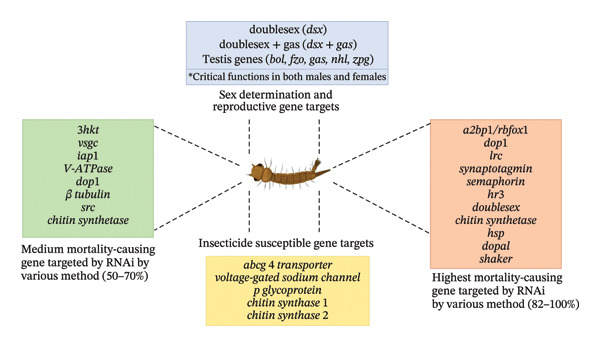
Mosquito genes targeted by RNA interference (RNAi) for vector control.

Mosquito control has been exploited by targeting of genes that are involved in the development of mosquito or the transmission of the pathogen using a conserved mechanism known as RNAi for the silencing of the genes [74]. This entails the introduction of double‐stranded RNA (dsRNA) molecules that targets and degrades directly the complementary mRNA transcripts, which eventually leads to the supersession of gene expression [75]. This approach is used for targeting the genes responsible for malaria parasite development or mosquito survival within the mosquito host [76]. RNAi is employed to silence genes involved in mosquito reproduction, development, or immunity to reduce the mosquito population size or render mosquitoes less competent for transmitting malaria parasites [77, 78] (Figure 4).

RNAi‐mediated silencing of mosquito genes involved in survival, insecticide susceptibility, sex determination, and reproduction can reduce vector populations and interrupt malaria transmission. Target genes include both male‐ and female‐relevant genes, such as *doublesex (dsx)* and *zero population growth (zpg)*, which play essential roles in sexual development and female germline maintenance. Silencing of these genes through dsRNA processing by Dicer and subsequent RISC‐mediated mRNA degradation can result in reduced fertility, increased mortality, or impaired parasite development, thereby contributing to effective malaria vector control [76]. Although several RNAi targets have been investigated for inducing male sterility or lethality, many genes such as *doublesex* and *zpg* have critical functions in females as well. This strategy is promising as targeting such genes can leads to impairment of female fertility, which can result into reduction of transmission or suppression of the population [74].

The potential malaria transmission can be disrupted by targeting essential genes that are involved in generation of embryo, egg development, or immune responses in mosquito by introducing dsRNA molecules [79]. This can make accessible the impact of gene knockdown on the mosquito physiology, immunity, and susceptibility [80]. Some studies have used RNAi‐mediated gene silencing in mosquitoes to target many genes in the developmental stage of the mosquito, and some of the larvae stage genes, which include ataxin 2‐binding protein and chitin synthetase, are effective when silenced with various delivery methods [76]. Targeting prophenoloxidase III and cecropin B gene through suppression in the pupae stage shows irregularities through microinjection and in adults, ataxin 2‐binding protein and dopamine 1 receptor genes give a high mortality through the yeast delivery method, showing the efficiency of the methods across mosquito species. Also, genes responsible for the reproduction of mosquito such as vitellogenin or eggshell proteins when silenced can disrupt mosquito fecundity and put a limitation to the growth of the population [76, 81]. Immune‐related genes such as PRRs or signaling molecules can be targeted to mitigate mosquito responses to malaria parasite and reduce vector competence [21].

As promising as this RNAi‐based strategies are for mosquito control, several challenges need to be overcome for its efficacy; this includes the delivery methods, off‐target effects, and persistence of gene silencing effects [82–84]. The proper delivery of dsRNA molecules to mosquito tissues is crucial for efficient gene silencing [83, 85]. Off‐targets effect that results from silencing unintended genes can lead to unintended outcome. Proper optimization of the delivery methods and the accurate selection of target genes can yield an efficient gene‐silencing approach to mosquito population [86].

### 1.6. Self‐Limiting Genetic Strategies for Mosquito Control

Self‐limiting mosquito control strategies are unique because their effect does not persist in the environment [87]. In contrast to self‐sustaining gene drive systems, they require continuous releases of treated or transgenic insects, with no spread in the population once releases cease. These systems can still be self‐limiting gene drives with biased inheritance, which can reduce the necessary release size [88]. These approaches are a lower‐risk alternative for vector control because they are noninvasive and reversible and can be adopted in locations with ecological and regulatory concerns.

The release of insects carrying a dominant lethal (RIDL) system is a very established self‐limiting approach where mosquitoes are engineered to carry a dominant lethal gene that will result in the death of offsprings in the absence of a tetracycline repressor protein. The male RIDL‐modified mosquitoes are released to mate with the wild‐type female mosquitoes; with this, their offspring will not survive, thereby leading to reduction of the population size over successive generations [89]. This strategy has been deployed in *Aedes aegypti* to control dengue and other arboviruses, which yielded suppression in several field trials [90]. This approach has also been adopted in *Anopheles* mosquitoes to show their promising potential for malaria control. This system is promising in reducing vector density because RIDL‐induced lethality is self‐limiting and does not spread through populations. However, there are some challenges that make this technology difficult to adopt, and these include the technical difficulties involved in genetic manipulating and mass rearing of RIDL strains for *A. gambiae* even though RIDL has been developed for the Asian malaria vector *A. stephensi.* This is due to the complex nature of *A. gambiae,* the mating competitiveness of transgenic males, the need for large‐scale release, and the acceptability of this strategy in African communities [91].

Another recent self‐limiting genetic approach is the precision‐guided sterile insect technique (pgSIT) which operates with the combination of CRISPR/Cas9‐mediated gene editing with classical SIT [92]. The genetic construct involved are developed in a way to disrupt the genes responsible for fertilization or sex determination to produce sterile males when released. Unlike the homing gene drives, this approach does not operate on the basis of biased inheritance or autonomous spread, and the effect of its suppression is restricted to the cohort that is being released. This approach has been successfully designed for *Aedes* and *Drosophila*; however, no *Anopheles* species yet. Although pgSIT is efficient in the laboratory and cage studies, it has not been employed in a field setting [93]. The limitations to this approach include a need for repeated release and the lack of drive dynamics that may pose a challenge to scalability compared with gene drive strategies [94].

### 1.7. Advantages and Limitation in Genetic Malaria Vector Control

Genetic strategies for malaria vector control offer transformative potential but are accompanied by distinct technical, ecological, and operational challenges that must be carefully considered.

The gene drive approaches, especially the ones that make use of CRISPR/Cas9 mechanisms, are effective in population suppression within the mosquito population. They are sustainable and require minimal release efforts, which makes them promising for malaria vector control [53]. The concerns of ecological impacts and regulatory issues need to be considered for responsible deployment of this approach to mitigate potential risks [57]. The *Wolbachia*‐based strategies, even though are effective in the reducing vector competence and interrupting malaria transmission cycles [73], its deployment to the field has shown inconsistent results [65]. The RNAi‐mediated strategies, which are suitable for targeting essential genes to disrupt malaria transmission [95], face the limitation of effective delivery and off‐target effect. The concern of introducing genetically modified mosquitoes to the ecosystem which could have unexpected effect on nontarget species and the ecosystem is the limitation to the adoption of these approaches, which needs proper intervention [96]. The implementation of subsequent monitoring programs to track the changes in the ecosystem, mosquito populations, and nontarget species should be put in place before adoption [97].

RIDL and pgSIT, the self‐limiting genetic approaches, show a great potential than the gene drive system and could be more acceptable in a region that values ecological and ethical concerns; however, the dependent on repeated releasement may pose a challenge of scalability and cost‐effectiveness in regions with high transmission rate [93, 98].

The immune regulator mosGILT has shown potential relevance in blocking malaria transmission although studies are still exploring this approach. Future research could focus on evaluating multiple immune pathways and not target a single gene to better understand how different immune mechanisms contribute to parasite suppression.

## 2. Conclusion

This review explores the relevance of mosquito immunity and genetic approaches in malaria vector control. Gene drive technologies are promising but raise ecological and regulatory concerns. While *Wolbachia*‐based interventions have been successful in arboviral control, their application in *Anopheles* mosquitoes has produced inconsistent results. RNA interference and symbiont‐based strategies have advanced the understanding of mosquito–parasite interactions; however, their effectiveness varies with limitations. Self‐limiting genetic strategies show a great potential than the gene drive system and could be more acceptable; however, the dependent on repeated releasement may pose a challenge of scalability and cost‐effectiveness in regions with high transmission rate. Therefore, designing genetic tools with ecological and community considerations remain essential for developing sustainable malaria control strategies.

## Author Contributions

Eniola D. Adedoyin: conceptualization, writing–original draft, and visualization.

Suleiman Zakari and Olubanke O. Ogunlana: formal analysis, investigation, and writing–review and editing.

Wisdom D. Clenclay, Shalom N. Chinedu, and Emeka E. J. Iweala: writing–review and editing and supervision.

## Funding

This work received no funding in terms of grants and other monetary gifts.

## Disclosure

The authors confirm that this submission is not under consideration and has not been published in any other journal—in whole or in part. This manuscript is an original work that has not been published or submitted elsewhere. All authors have approved the manuscript for publication.

## Conflicts of Interest

The authors declare no conflict of interest.

## Data Availability

No new data were generated in this study.
